# Resilienz deutscher Kliniken in Amok- und Terrorlagen: Ergebnisse einer onlinebasierten Umfrage deutscher Notaufnahmen

**DOI:** 10.1007/s00103-023-03752-x

**Published:** 2023-08-21

**Authors:** Marvin Schulz, Jörg-Wilhelm Oestmann, Thorsten Schütz

**Affiliations:** 1 9./Fallschirmjägerregiment 31, Seedorf, Deutschland; 2 Klinik für Radiologie, CC06, Charité, Campus Mitte, Charitéplatz 1, 10117 Berlin, Deutschland; 3 Führung Streitkräfte (FüSK) San 1, Bundesministerium der Verteidigung, Berlin, Deutschland

**Keywords:** KRITIS, Terrorismus, Resilienz, KAEP, Krankenhaus, Critical infrastructure, Terrorism, Resilience, Hospital emergency preparedness planning, Clinic

## Abstract

**Hintergrund:**

Krankenhäuser müssen sich als kritische Infrastruktur (KRITIS) auf terroristische Anschläge sowie Amoklagen einstellen und mögliche Schadensabwehrmaßnahmen vorbereiten, da sie leicht zu treffende Ziele mit hoher Symbolkraft darstellen.

**Methode:**

In der vorliegenden Arbeit wurde die terroristische Bedrohungslage anhand einer Auswertung der Terrorist Event Database (TED) für den Zeitraum 1970–2017 quantifiziert. Darüber hinaus wurden die Bedrohungsperzeption sowie die getroffenen Vorbereitungen mittels einer Online-Umfrage unter den Leitenden deutscher Notaufnahmen erfasst.

**Ergebnisse:**

Die Auswertung der TED zeigt international einen Anstieg der terroristischen Angriffe auf medizinische Einrichtungen mit Schwerpunkt in Ländern im (Bürger‑)Kriegszustand sowie in den USA. Der Einsatz von Explosivstoffen führte im untersuchten Zeitraum zu einer hohen Anzahl an Verletzten und Toten. Die Online-Befragten empfanden eine Vorbereitung der Klinik auf terroristische Szenarien in ihren Krankenhausalarm- und -einsatzplänen (KAEP) überwiegend als wichtig. In den bestehenden KAEP waren entsprechende Maßnahmen dennoch häufig nicht etabliert. Nur in 59,4 % wurde die Verletztenversorgung im Rahmen eines terroristischen Angriffs und nur in 34 % die Sicherung der Infrastruktur behandelt.

**Schlussfolgerung:**

Deutsche Kliniken sollten sich als kritische Infrastruktur sowie als mögliches Ziel terroristischer Anschläge verstehen und sich auf terroristische Bedrohungen vorbereiten. Viele günstig umzusetzende Maßnahmen sind in den Kliniken nicht ausgeschöpft und sollten zeitnah sowohl in den KAEP als auch in den Alltag integriert werden.

## Abstract (en)

**Background:**

As a critical infrastructure (CRITIS) and soft target with high symbolic power, hospitals have to prepare for terrorist attacks, rampages, and establish damage-prevention measures.

**Method:**

The terrorist threat situation was quantified using an evaluation of the terrorist event database (TED) from 1970 to 2017. Additionally, the threat perception and precautionary measures were recorded by an online survey among the leaders of German emergency departments.

**Results:**

The TED-evaluation shows an international increase in terrorist attacks on medical facilities, especially in civil war countries and the USA. The use of explosives caused a large number of injuries and deaths during the examined time period. Most online respondents felt that preparing the clinic for terrorist scenarios in their hospital emergency preparedness planning was important. Suitable measures were, however, often not established in hospital emergency preparedness planning. Only 59% dealt with the care of the injured due to a terrorist attack and only 34% with securing the infrastructure.

**Conclusion:**

German clinics should consider themselves as critical infrastructure and potential targets of terrorist attacks and prepare themselves for terrorist threats. Many measures that could be implemented cheaply have not been utilized in the clinics and should be integrated promptly into both hospital emergency preparedness planning and daily routines.

## Einleitung

„Infrastrukturen im Allgemeinen und Kritische Infrastrukturen im Besonderen sind die unverzichtbaren Lebensadern moderner, leistungsfähiger Gesellschaften“ [1].

Krankenhäuser und medizinische Einrichtungen sind Teil dieser kritischen Infrastruktur (KRITIS) und neben ihrer vernetzten Stellung als Gesundheitsdienstleister auch bedeutsam im Rahmen der inneren Sicherheit [2]. Nach den Terroranschlägen in Madrid im Jahr 2004 wurde die Sicherheit der KRITIS erstmalig auf die europäische Tagesordnung gesetzt [3].

Das Bundesministerium des Innern und für Heimat (BMI) hat mehrere Positionspapiere zu dieser Thematik herausgebracht. Insbesondere in der Broschüre „Nationale Strategie zum Schutz Kritischer Infrastruktur“ finden sich Definitionen, Zielsetzungen und Aufträge an sowohl staatliche als auch private Stellen. Als eine der Hauptaussagen wird hier hervorgehoben, dass es „eine Kernaufgabe staatlicher und unternehmerischer Sicherheitsvorsorge und zentrales Thema der Sicherheitspolitik“ ist, den Schutz dieser Infrastrukturen sicherzustellen [1]. Das BMI definiert terroristische Bedrohungen und Naturgefahren als die 2 hauptsächlichen Gefährdungsursachen. Um den Schutz der KRITIS in Deutschland zu gewährleisten, werden insbesondere Ziele für die Bereiche Prävention, Reaktion und Nachhaltigkeit formuliert.

Die maßgebliche Verantwortung für die Vorgaben und die Umsetzung der KRITIS-Strategie tragen der Bund und die Länder. Da 80 % der KRITIS jedoch ausschließlich von privaten bzw. privatisierten Unternehmen betrieben werden, sind diese als zentrale Akteure über ihre Betreiberverantwortung intensiv eingebunden [4]. Die Vulnerabilität der KRITIS muss erkannt und minimiert werden, um die Widerstandsfähigkeit der Gesellschaft zu erhöhen [5]. Dabei müssen die Kosten für die Eigentümer und Betreiber an die Risiken angepasst und zumutbar bleiben [3].

Die Fragen, ob eine Vorbereitung auf einen physischen terroristischen Anschlag auf deutsche Kliniken notwendig ist und inwieweit die Kliniken im Rahmen ihres Krankenhausalarm- und -einsatzplans (KAEP) auf ein solches Szenario vorbereitet sind, wurden im Rahmen der vorliegenden Arbeit untersucht.

Hierbei wurden nur physische terroristische Angriffe bzw. Angriffe im Rahmen einer Amoklage einbezogen. Cyberterrorismus wurde explizit nicht betrachtet und dementsprechend im Fragebogen ausgeschlossen.

## Methoden

Es erfolgten 2 umfangreiche Literaturrecherchen beginnend im Jahr 2019 zu den Themen „kritische Infrastruktur“ sowie „moderner Terrorismus, Anschläge und Entwicklungen in Europa“. Zusätzlich wurden begleitend zum Projekt mehrfach Literaturabfragen der elektronischen Datenbanken von Pubmed, eRef und der virtuellen Bibliothek der Bundeswehr durchgeführt. Suchparameter hierbei waren Kombinationen der Schlagworte KRITIS, Krankenhaus, Terror, Amok, Anschlag, Resilienz, Kliniken.

Zusätzlich wurde die Terrorist Event Database (TED; [6]) genutzt, um Anzahl sowie Charakteristika und Folgen terroristischer Ereignisse (exklusive Cyberterrorismus) gegen medizinische Gebäude weltweit von 1970 bis 2017 zu analysieren. Es handelt sich hierbei um eine am Fraunhofer-Institut für Kurzzeitdynamik, Ernst-Mach-Institut (EMI) entwickelte, nicht öffentliche Datenbank, welche weitestgehend alle terroristischen Ereignisse weltweit beinhaltet. Für die vorliegende Arbeit wurden in der TED lediglich Einträge mit folgenden Zielsystemen gewählt: medizinische Gebäude (Krankenhäuser, Pflege), medizinische Ziele (unbekannt/andere), medizinisches Personal (Ärzt*innen, Pfleger*innen, Sanitäter*innen), medizinische Fahrzeuge (Rettungswagen). Für eine bessere Aussagekraft bezüglich der Bedrohungslage von Kliniken wurde aus dieser Auswahl die abschließende Analyse lediglich mit dem Zielsystem „medizinische Gebäude“ durchgeführt.

Zur Durchführung der eigenen Umfrage wurde ein Online-Fragebogen (SoSci Survey) genutzt. Die Auswahl der Items erfolgte unter Einbeziehung mehrerer Fokusgruppen (zivile Ärzt*innen, Sanitätsoffizier*innen, Polizist*innen, Feuerwehrleute, Rettungsdienstpersonal und andere Spezialist*innen). Die entworfenen Fragen wurden anhand des „Fragebewertungssystems“ [7] mittels Checkliste einzeln einer Qualitätsprüfung unterzogen.

Anschließend erfolgte ein doppelstufiger onlinebasierter Prätest. Hierbei wurde der Fragebogen in seiner damaligen Form über die Online-Plattform zur Reevaluation an eine Gruppe von 20 Personen verschiedener Fachrichtungen (Medizin, Mathematik, Pädagogik etc.) verteilt. Der onlinebasierte Prätest erfolgte in 2 aufeinanderfolgenden Phasen mit wechselnden Bearbeitungsgruppen. Im Anschluss wurden 10 Face-to-Face-Interviews des Fragebogens ebenfalls mit Personen verschiedener Fachrichtungen durchgeführt. Abschließend erfolgte intern eine technische Prüfung des Online-Umfragebogens zur Prüfung der korrekten Datenein- und -ausgabe sowie der korrekten PHP^1^-Funktionen. Nach Beendigung dieser Prüfvorgänge wurde der Fragebogen über den E‑Mail-Verteiler eines gemeinsamen Notaufnahmeverzeichnisses der Deutschen Gesellschaft Interdisziplinäre Notfall- und Akutmedizin (DGINA) und der Deutschen Interdisziplinären Vereinigung für Intensiv- und Notfallmedizin (DIVI) versandt. Ziel dieses Verzeichnisses ist die Erfassung möglichst aller Notaufnahmen in Deutschland, inklusive eines persönlichen Ansprechpartners. Es soll u. a. einer besseren Vernetzung notfallmedizinischer Abteilungen dienen.

Der Versand des Fragebogens erfolgte am 25.08.2021. Die Umfrage endete nach 37 Tagen am 30.09.2021. 2 Wochen nach Versand des Fragebogens erhielten die Teilnehmenden eine Erinnerungs-E-Mail. Die Beantwortung des Fragebogens sollte explizit nur durch die ärztliche oder pflegerische Leitung der Notaufnahme durchgeführt werden.

Der Online-Fragebogen beinhaltete verschiedene Themenblöcke: Im ersten Fragenblock wurden die Befragten gebeten, ihre Haltung zu verschiedenen Aussagen (z. B. „Einen terroristischen Angriff auf ein deutsches Krankenhaus halte ich in den nächsten 10 Jahren für möglich“) auf einer Likert-Skala von 0 (*Lehne die Aussage vollkommen ab*) bis 5 (*Stimme der Aussage vollkommen zu*) einzuordnen. Im zweiten Fragenblock wurden Fragen zum Inhalt des KAEP gestellt, die mittels binärer Auswahlmöglichkeiten (*Ja/Nein*) beantwortet wurden. Im letzten Fragenblock wurde nach der Zusammenarbeit mit der Polizei und der Durchführungsfrequenz von Übungen gefragt. Teilweise konnten Fragen durch Freifeldeingaben beantwortet werden.

Die statistische Auswertung der Daten erfolgte in Excel.

## Ergebnisse

### Terrorist Event Database

Zum Zeitpunkt des Zugriffes auf die Datenbank am 26.06.2019 waren 178.000 Ereignisse, 214 Länder, 99 Taktiken (Anschlagsart), 2663 Terrorgruppen und 290 Ziele (unterteilt in Kategorien, z. B. zivil, öffentlich) hinterlegt. Im Zeitraum von 1970 bis 2017 waren weltweit 691 Anschläge auf medizinische Gebäude mit 860 Todesopfern und 1829 Verletzten zu verzeichnen (siehe Auswahl in Tab. 1). Die meisten Anschläge erfolgten mit konventionellen und unkonventionellen Sprengvorrichtungen (*n* = 348; 50,4 %), gefolgt von Feuer oder Brandvorrichtungen (*n* = 176; 25,5 %) und Handfeuerwaffen (*n* = 82; 11,9 %). Die Taktiken *Suicide Bomber*^2^ (*n* = 18; 2,6 %) und *Vehicle Borne Improvised Explosive Device*^3^ (*n* = 18; 2,6 %) waren seltener.

**Table 1:** 

Taktik (Anschlagsart)	Anschläge	Todesopfer	Verletzte
Armed_Attack_(Explosives)	4	13	20
Armed_Attack_(Fire_or_Firebomb)	176	24	10
Armed_Attack_(Firearms)	82	106	80
Barricade_Hostage_(Firearms)	3	16	41
Barricade_Hostage_(Knives_&_Sharp_Objects)	1	19	26
Bombing_(Explosives)	348	452	1328
Bombing_(Suicide)	18	98	97
Bombing_-_Car_(Explosives)	18	63	218
Hijacking_(Firearms)	1	9	0
Kidnapping_(Firearms)	4	0	1
*Summe*	655	800	1821

Durch die Anschläge mittels konventioneller und unkonventioneller Sprengvorrichtungen kam es in dem oben genannten Zeitraum zur höchsten Anzahl an Verletzten (*n* = 1328; 72,6 %^4^) und Todesopfern (*n* = 452; 52,6 %). Angriffe mit Feuer und Brandvorrichtungen verursachten trotz der prozentual hohen Anzahl an Anschlägen im Vergleich eher wenige Verletzte (*n* = 10; 0,5 %) und Todesopfer (*n* = 24; 2,8 %). Die verhältnismäßig geringe Anzahl an Anschlägen mittels *Vehicle Borne Improvised Explosive Device* führte zu vielen Verletzten (*n* = 218; 11,9 %) und Todesopfern (*n* = 63; 7,3 %) in dem oben genannten Zeitraum.

**Figure 1:**
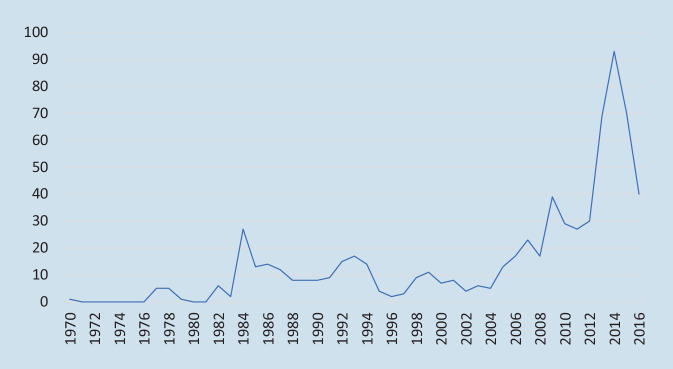

Es lässt sich im Verlauf eine Zunahme der weltweiten Anschläge auf medizinische Gebäude mit einem Höhepunkt im Jahr 2014 (*n* = 93 Anschläge) feststellen (Abb. 1). Von den 691 terroristischen Anschlägen auf medizinische Einrichtungen in dem untersuchten Zeitraum ereigneten sich allerdings nur 2 Vorfälle in Europa, davon einer in Frankreich und einer in Griechenland. Bei beiden gab es weder Verletzte noch Tote. Die meisten Anschläge fanden in den USA (*n* = 218; 31,5 %) statt. Hier wurde jedoch von vergleichsweise wenigen Todesopfern (*n* = 7; 0,8 %) und Verletzten (*n* = 26; 1,4 %) berichtet. Insbesondere in Ländern mit Bürgerkriegszuständen kam es zu vielen Toten und Verletzten bei Angriffen auf medizinische Infrastrukturen. So wurden zum Beispiel im Irak 13,9 % der Anschläge weltweit verzeichnet (*n* = 96). Hierbei kam es zu 196 Toten (22,8 %) und 676 Verletzten (37 %).

### Online-Umfrage

Die Umfrage wurde über den E‑Mail-Verteiler des Notaufnahmeverzeichnisses der DGINA und DIVI an 859 Empfänger gesendet. Davon kamen 47 E-Mails als unzustellbar zurück, woraufhin nach Recherche der aktuellen E‑Mail-Adresse ein erneuter Versand versucht wurde. Insgesamt wurde der Fragebogen von 106 Personen abgeschlossen. Dies entspricht einer Rückläuferquote von 12,3 %. Bei einer geschätzten Zahl von ca. 1000 zentralen Notaufnahmen deutschlandweit im Jahr 2009 [8] wurden somit die Angaben von über 10 % aller Notaufnahmen eingeschlossen.

Bei den ärztlichen oder pflegerischen Leitenden der Notaufnahmen, die den Online-Fragebogen beantworteten, zeigte sich folgende Verteilung der Arbeitsstätten: Regelversorgung (*n* = 40; 37,7 %), Schwerpunktversorgung (*n* = 47; 44,3 %), Maximalversorgung (*n* = 9; 8,5 %), Bundeswehrkrankenhaus (*n* = 1; 0,9 %), Universitätsklinik (*n* = 8; 7,5 %) und andere (*n* = 1; 0,9 %).

### Auswertung der Relevanzwahrnehmung der Thematik in deutschen Kliniken

Die Aussage: „Einen terroristischen Angriff auf ein deutsches Krankenhaus halte ich in den nächsten 10 Jahren für möglich“, wurde von 74,5 % der Befragten mit einem Likert-Skalenwert von 3 oder höher beantwortet. Die Aussage: „Deutsche Krankenhäuser sollten sich in ihren Krankenhausalarm- und -einsatzplänen (KAEP) medizinisch auf einen terroristischen Angriff und die Besonderheiten der Versorgung hier auftretender Verletzter vorbereiten“, wurde ebenso wie die Aussage: „Deutsche Krankenhäuser sollten sich in ihren Krankenhausalarm- und -einsatzplänen (KAEP) infrastrukturell (z. B. durch Schließen von Zugängen) auf einen terroristischen Angriff mit der eigenen Klinik als Ziel vorbereiten“, von über der Hälfte der Befragten mit einem Likert-Skalenwert von 5 bewertet. Bezüglich der medizinischen Vorbereitung beantworteten 65,1 % der Teilnehmenden die Frage mit einem Likert-Skalenwert von 5 und bezüglich der infrastrukturellen Vorbereitung 55,7 %. Die Aussage: „Die Möglichkeit einer terroristischen Bedrohung sollte bei baulichen Neuplanungen von Kliniken stärker berücksichtigt werden“, wurde von 46,2 % der Befragten und die Aussage: „Eine Ausbildung des Klinikpersonals, insbesondere des Sicherheitsdienstes, in Bezug auf Terrorbedrohungen sollte durchgeführt werden, um die Sicherheit der eigenen Klinik in terroristischen Bedrohungslagen zu erhöhen“, wurde von 50 % der Befragten mit einem Likert-Skalenwert von 5 beantwortet.

### Auswertung der im KAEP hinterlegten Maßnahmen

Die Frage, ob in dem eigenen KAEP das Thema der Verletztenversorgung im Falle eines terroristischen Angriffs in ihrem Einzugsgebiet berücksichtigt wird, wurde mehrheitlich bejaht (Ja = 59,4 %; Nein = 40,6 %). Die Frage, ob der KAEP die Sicherung der Infrastruktur beinhaltet, wenn der Verdacht auf einen gerichteten terroristischen Angriff auf die eigene Klinik besteht, wurde mehrheitlich verneint (Nein = 66 %; Ja = 34 %).

Es ergaben sich dabei wesentliche Unterschiede je nach Versorgungsstufen: So wurde die Verletztenversorgung in Terrorlagen bei 82,4 % der Maximalversorger und Universitätskliniken berücksichtigt, wohingegen dies nur bei 53,2 % der Schwerpunktversorger und 57,5 % der Regelversorger der Fall war. Die Sicherung der Infrastruktur im Angriffsfall wurde bei 58,8 % der Maximalversorger und Universitätskliniken behandelt, jedoch nur bei 27,7 % der Schwerpunktversorger und 30 % der Regelversorger.

**Figure 2:**
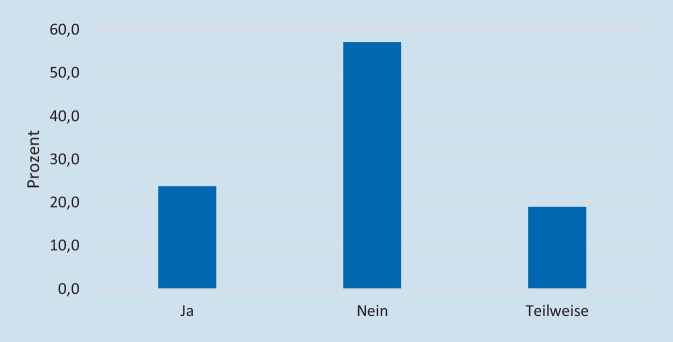

65,1 % der Kliniken gaben an, dass in ihrem KAEP Maßnahmen benannt sind, um Zugänge zur Klinik zu verschließen und/oder zu kontrollieren. Maßnahmen zur Kontrolle von eintreffenden Verletzten und/oder eintreffenden Rettungsmitteln waren in 37,7 % (*n* = 40) vorgesehen, in 62,3 % (*n* = 66) nicht eingeplant. In 23,8 % (*n* = 25) der Kliniken führen die Mitarbeitenden im täglichen Dienst personalisierte Identifikationsausweise mit sich, in 19 % (*n* = 20) werden die Ausweise teilweise mitgeführt, in 57,1 % (*n* = 60) ist das nicht der Fall (Abb. 2). Die Frage bezüglich der Identifikationsausweise wurde von einem Teilnehmenden an der Studie nicht beantwortet, daher beziehen sich die Prozentangaben auf 105 von 106 Teilnehmenden.

### Auswertung der menschlichen Faktoren

Einen durchgängig mit Personal präsenten Sicherheitsdienst gab es nur in 28,6 % (*n* = 30) der Kliniken. In der Gruppe der Maximalversorger und Uniklinken war dies signifikant (*p* = 0,000007) häufiger (76,5 %) als in der Schwerpunktversorgung (26,1 %) und Regelversorgung (10 %). Die Frage nach dem Sicherheitsdienst wurde von einem Teilnehmenden an der Studie nicht beantwortet, daher beziehen sich die Prozentangaben auf 105 von 106 Teilnehmenden.

**Figure 3:**
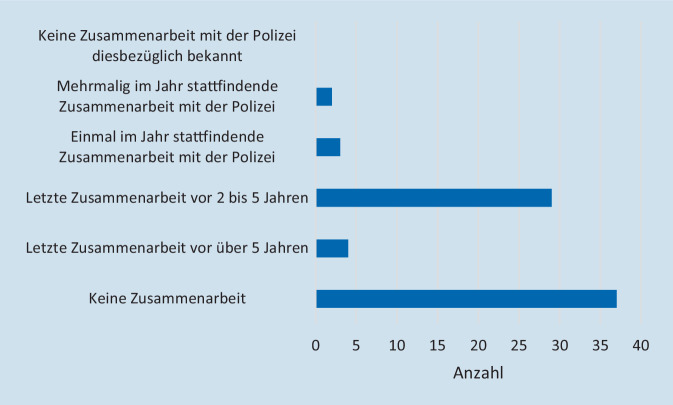

Eine Zusammenarbeit der eigenen Klinik mit der Polizei bei der Weiterentwicklung des KAEP bezüglich terroristischer Bedrohungslagen wurde in 49,3 % (*n* = 37) verneint (Abb. 3). Bei 5,3 % (*n* = 4) hatte eine letztmalige Zusammenarbeit vor über 5 Jahren, bei 38,7 % (*n* = 29) vor 2–5 Jahren stattgefunden. 4 % (*n* = 3) gaben eine einmalige und 2,7 % (*n* = 2) eine mehrmalige Zusammenarbeit pro Jahr an. Insgesamt beantworteten nur 75 von 106 Teilnehmenden diese Frage. In 41,9 % (*n* = 26) wurde der Polizei Kenntnis von den Inhalten des KAEP der Klinik gegeben. Diese Frage wurde von 62 der 106 Teilnehmenden beantwortet.

**Figure 4:**
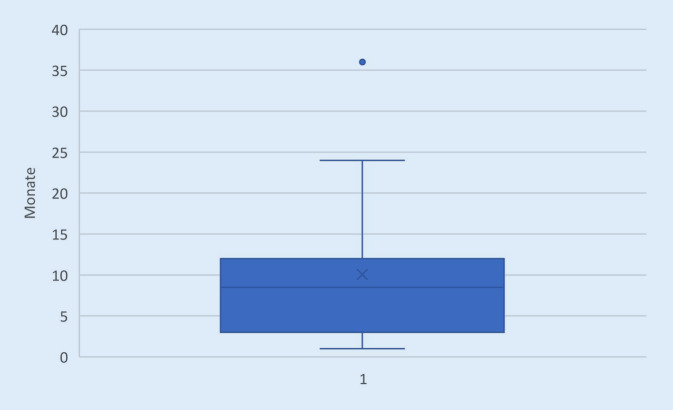

Gemittelt 27 % (Standardabweichung (SD) ± 24) der Klinikmitarbeitenden kannten nach Einschätzung der Beantwortenden den Inhalt des KAEP. Die letzte Überprüfung des KAEP hatte im Mittel vor 10 Monaten stattgefunden (SD ± 9; Abb. 4). Der regelmäßige Überprüfungszyklus des KAEP wurde mit 15 Monaten (SD ± 9 Monate) angegeben.

**Figure 5:**
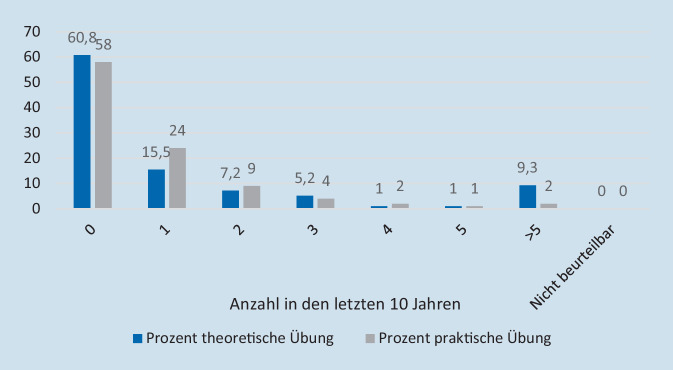

60,8 % (*n* = 59) der Befragten hatten in den letzten 10 Jahren keine theoretische und 58 % (*n* = 58) keine praktische Übung durchgeführt (Abb. 5). Die Frage nach den theoretischen Übungen beantworteten 97 und die nach praktischen Übungen 100 der Teilnehmenden. Bei durchgeführten Übungen wurde die Polizei größtenteils weder als Teilnehmender (69,6 %; *n* = 32) noch als Beobachtender/Beurteilender (74,5 %; *n* = 35) einbezogen. Bei den Übungen wurde nur in 37,2 % (*n* = 16) ein Verschluss und/oder die Kontrolle von Zugängen zum Krankenhaus geübt.

## Diskussion

Die Auswertung der TED zeigt, dass Anschläge gegen medizinische Gebäude als weiche Ziele zunehmen. Auch zukünftig ist damit zu rechnen, dass medizinische Zentren für Terrorist*innen attraktive Ziele darstellen, da die von den Akteuren erwünschte Demoralisierung der Bevölkerung durch solche Taten als hoch einzustufen ist [5, 9]. Ergänzend kann hierbei auch die Nutzung solcher terroristischen Anschläge im Rahmen einer „hybriden Bedrohung“ bei zwischenstaatlichen Konflikten einbezogen werden [10]. Laut BMI handele es sich dann um hybride Bedrohungen, wenn „nicht legitime Methoden der Einflussnahme aus dem Ausland eingesetzt werden, ohne dass ein Krieg erklärt wird“ [11]. Die Definition und Auslegung des Begriffs werden in der Wissenschaft jedoch aktuell noch breit diskutiert [12].

Die hohen Anschlagszahlen in den USA unterstreichen, dass nicht nur vor dem Hintergrund kriegs- oder bürgerkriegsähnlicher Zustände eine reale Gefahr besteht. Das liberale Waffenrecht, die lückenhafte gesundheitliche Versorgung und langjährige gesellschaftliche Konflikte um Abtreibungsregelungen [13] werden zur Erklärung der dortigen Häufung herangezogen. Der Krieg in der Ukraine, parallele Cyberangriffe, Sabotageakte an Pipelines und Datenleitungen zeigen jedoch auch die zunehmende Dynamik der Gewalt hier in Europa, die unsere Krankenhäuser nicht ignorieren können. Da in einem solchen Szenario der Schutz der Klinik nicht zwingend zeitnah durch polizeiliche Einsatzkräfte geleistet werden kann, obliegt die Verantwortung der Organisation für Vorsorge und die Bewältigung der Frühphase bei den Krankenhäusern [14]. Daher sollte erwogen werden, diese Szenarien in KAEP in Deutschland einzupflegen und die Mitarbeitenden zusätzlich dafür zu sensibilisieren und zu schulen.

Solche Aspekte sollten bereits in die Neuplanung und Umstrukturierung von Krankenhäusern einbezogen werden [15]. Unterstützend sollte hierbei das „Handbuch Krankenhausalarm- und -einsatzplanung (KAEP)“ des Bundesamts für Bevölkerungsschutz und Katastrophenhilfe (BBK) hinzugezogen werden [16]. In den Bereichen der medizinischen Versorgung, infrastrukturellen Maßnahmen und der polizeilichen Zusammenarbeit wird das Thema einer terroristischen oder Amokbedrohung durch die Autor*innen aufgegriffen und mit Handlungsempfehlungen hinterlegt. Im Gegensatz dazu beschäftigt sich die Weltgesundheitsorganisation (WHO) in verschiedenen Veröffentlichungen zwar auch mit dem Thema der Resilienz im Gesundheitssystem, präsentiert das Thema der terroristischen Bedrohungen aktuell jedoch nur als kleine Randnotiz [17, 18].

Die Analyse der TED deutet darauf hin, dass das terroristische Vorgehen gegenüber Kliniken dem auf andere Ziele ähnelt. Anschläge mit Explosivstoffen sind besonders häufig und fordern viele Opfer [19]. Für diese Problematik wurde im Rahmen des Forschungsprojektes MULTISCHUTZ vom BBK, dem Fraunhofer EMI und Mehler Engineered Defence ein Bauteilsystem zum Schutz von Personen und Infrastruktur entwickelt [20].

Auch solche Maßnahmen sollten bei einer Neuplanung und Anpassung der bestehenden Verhältnisse zur „Härtung“ im Sinne einer Verstärkung des Gebäudes gegenüber physischen Angriffen bedacht werden. Denn die Krankenhäuser und insbesondere die Notfallaufnahmen zu sicheren Bereichen während einer bedrohlichen Lage zu machen, wurde bereits 2017 in den Ergebnissen des Konsensusgesprächs „Zusammenarbeit von Rettungskräften und Sicherheitsbehörden bei bedrohlichen Lagen“ aus dem wissenschaftlichen Arbeitskreis „Notfallmedizin der Deutschen Gesellschaft für Anästhesie“ formuliert [21]. Aber auch andere offene oder verdeckte Möglichkeiten der Sicherheitsarchitektur zur Verstärkung der Zugangskontrolle, Schaffung von Distanz, Einbeziehung von Deckung und Sichtschutz und Einrichten von Schutzräumen sollten in Betracht gezogen werden [22].

Ergänzend zu diesen infrastrukturellen Maßnahmen muss das Personal geschult werden, eine Gefährdungssituation zu erkennen, eine weiterführende Alarmierung zu initiieren und sich selbst und andere durch Einschließen und Suchen von möglichen Deckungen zu schützen [23].

Zusätzlich zu den genannten Maßnahmen muss aber im Falle einer terroristischen Bedrohung auch die Führungsorganisation der Klinik angepasst werden. Hierbei sollte „die operativ-taktische von der administrativ-organisatorischen Komponente“ getrennt werden [24].

Die Umfrage bestätigt, dass die Relevanz des Themas von den Befragten als überaus hoch eingeschätzt wird, aber bereits einfache Maßnahmen noch nicht im KAEP aufgenommen sind. Es wird deutlich, dass die Gruppe der Universitätskliniken und Maximalversorger im Vergleich zur Gruppe der Regel- und Schwerpunktversorger bereits mehr Maßnahmen im KAEP aufgeführt hat und zusätzlich auch häufiger einen Sicherheitsdienst beschäftigt. Dies geht einher mit größeren, aktiveren und sozial belasteteren Notaufnahmen in diesen Häusern. Die im KAEP aufgeführten Maßnahmen müssen jedoch auch jedem Mitarbeitenden bewusst sein und regelmäßig geübt werden, um im Ernstfall zu greifen [25].

Die in großen Teilen fehlende Koordination mit der Polizei ist besonders problematisch, da diese eine Kernaufgabe beim Management eines terroristischen Szenarios hat. Diese regelmäßigen Absprachen sollten auch auf andere öffentliche Behörden (Feuerwehr, Ämter, Sicherheitsdienste und ähnliche) erweitert werden [26].

## Fazit

Zusammenfassend lässt sich festhalten, dass Kliniken sich im Rahmen ihrer sicherheitspolitischen Verantwortung als kritische Infrastruktur der Bundesrepublik Deutschland auch auf terroristische Szenarien a) baulich, technisch und organisatorisch vorbereiten, b) die Koordination mit der Polizei verbessern und c) ihre getroffenen Maßnahmen regelmäßig trainieren und reevaluieren sollten.

## Footnotes

1 „Personal Home Page Tools“, eine Skriptsprache hauptsächlich zur Erstellung von Webanwendungen.

2 Typischerweise Sprengstoffgürteleinsatz mit Selbstmordfolge.

3 Sprengstoffanschlag mit einer unkonventionellen Sprengvorrichtung verbracht mittels Fahrzeugs.

4 Bei den prozentualen Angaben zu Verletzten und Toten handelt es sich um den Anteil aller Verletzten bzw. Toten durch Anschläge auf medizinische Gebäude in diesem Zeitraum.
